# Impact of Mediated Intimate Interaction on Education: A Huggable Communication Medium that Encourages Listening

**DOI:** 10.3389/fpsyg.2016.00510

**Published:** 2016-04-19

**Authors:** Junya Nakanishi, Hidenobu Sumioka, Hiroshi Ishiguro

**Affiliations:** ^1^Intelligent Robotics Laboratory, Graduated School of Engineering Science, Osaka UniversityToyonaka, Japan; ^2^Hiroshi Ishiguro Laboratory, Advanced Telecommunication Research Institute InternationalKyoto, Japan

**Keywords:** listening, child education, huggable communication medium, mediated intimate interaction, mental states, classroom communication

## Abstract

In this paper, we propose the introduction of human-like communication media as a proxy for teachers to support the listening of children in school education. Three case studies are presented on storytime fieldwork for children using our huggable communication medium called Hugvie, through which children are encouraged to concentrate on listening by intimate interaction between children and storytellers. We investigate the effect of Hugvie on children's listening and how they and their teachers react to it through observations and interviews. Our results suggest that Hugvie increased the number of children who concentrated on listening to a story and was welcomed by almost all the children and educators. We also discuss improvement and research issues to introduce huggable communication media into classrooms, potential applications, and their contributions to other education situations through improved listening.

## 1. Introduction

Communication with others is an important process for acquiring generic knowledge in society, such as language, communication skills, and social manners. After learners receive and interpret the information presented by caregivers or teachers, they sometimes acquire new knowledge and skills based on feedback. Obviously, a learner's ability for information comprehension is fundamental in the initial learning phase to acquire generic knowledge.

Listening is one such crucial skill, especially in school education since the information that must be learned is generally provided verbally. For example, 68% of the class time in German primary school classes and 53% in U.S. college students is spent listening (Bohlken, 1999; Imhof and Weinhard, 2004). However, investigations have reported that many first graders in several countries start school unprepared for learning, including an inability to listen during class lessons (McClelland et al., 2000; Rimm-Kaufman et al., 2000; Sakakihara, 2010). Two other studies reported that at most only half of kindergarteners have mastered the basic skills that are involved in regulating behavior, including paying attention, following instructions, and controlling inappropriate actions (McClelland et al., 2000; Rimm-Kaufman et al., 2000). In Japan, this is called the first-grader problem (Sakakihara, 2010), which denotes that teachers assigned to first grade face teaching obstacles, because an increasing number of children suffer from such behavioral problems as being noisy, leaving their seats, and disrupting class activities. The Tokyo metropolitan board of education surveyed 1313 Tokyo public primary schools in 2009 and discovered such problems in about one-quarter of the schools. Not surprisingly, many studies have reported that such classroom behavior problems negatively influence student performance in reading, writing, and math (Klein, 2002; Lutz and Intermediate Unit, 2003; Spira and Fischel, 2005; Miles and Stipek, 2006; Bub et al., 2007; McClelland et al., 2007; Raver et al., 2008; Bulotsky-Shearer and Fantuzzo, 2011). This problem must be solved to avoid impeding children's development.

Teachers and researchers have addressed the development of a curriculum for school readiness that includes listening training (Brigman and Webb, 2003; Webster-Stratton and Reid, 2004; Denham, 2006). For example, the *Incredible Years Child Training Program* guides children in learning how to make friends and follow school rules, how to listen, wait, avoid interruptions, and quietly raise their hands to ask questions through practical training and small group discussions (Webster-Stratton and Reid, 2004). Unlike specific training, using supportive systems that improve the classroom's listening environment allows teachers to bypass the training time for school readiness because these systems can support children's listening in parallel with lessons, allowing teachers to devote more class time to regular lessons. For example, the *sound field amplification system* (Millett, 2008; Dockrell and Shield, 2012), which offers the possibility of immediately minimizing the impact of poor classroom acoustics on student learning, projects the teacher's voice so that children will have a better opportunity to clearly hear his/her instructions. This system does not reduce exposure to external sound sources. But importantly, raising the volume of the teacher's voice increases the speech signal levels relative to the levels of other sound sources. The impact of these systems was expanded to support children with hearing loss and to meet the recommended acoustical standards for noise levels and reverberation times. They also facilitate children's ability to discriminate words and spoken language more accurately and achieve better standardized test scores in early literacy and statistically and significantly improve attention, communication, and classroom behavior ratings (see Millett, 2008 for a review).

Although they successfully provided opportunities to acquire listening skills by improving the external conditions of classrooms, they do not help students prepare their own internal states for listening. Human mental states are important in the educational curriculum for readiness to learn, including listening (Raver and Knitzer, 2002; Brigman and Webb, 2003; Webster-Stratton and Reid, 2004; Denham, 2006; Thompson and Raikes, 2007) because they influence our ability for self-control (Baumeister and Heatherton, 1996). Stress and anxiety make it difficult for people to control themselves and concentrate on speakers (Vogely, 1998). This is a serious problem for children due to their limited ability to exercise self-control. Actually children, especially first graders, often feel stress in their school environment, relationships with classmates, and lessons (Fabian and Dunlop, 2007; Wong, 2015). Systems that support both the internal and external conditions of listeners must be developed.

In this context, we focus on social interactions where people touch each other, such as a caregiver holding a child and reading a story with a picture book to her/him. Such interactions have two advantages for encouraging children to concentrate on listening. First is the impact of the tactile channel on stress reduction, which is one known effect of interpersonal touch (Gallace and Spence, 2010). Unlike other methods for decreasing stress by visual or auditory stimulation (Katcher et al., 1984; Pelletier, 2004; Labbé et al., 2007), tactile stimulation reduces stress without disturbing the audiovisual information provided by speakers in typical lectures. We can listen to and look at a lecture while touching something; however, that is difficult while listening to or looking at others. Second is the intimate distance shared by a speaker and listener. Such distance easily draws the listener's attention to the speaker's voice because it might be the strongest stimuli among others, as in sound field amplification systems (Millett, 2008; Dockrell and Shield, 2012). Another problem is that teachers cannot simultaneously establish close interactions with every student. Even when just a few children crave physical contact from their teachers, physical contact limits the teacher's behaviors, such as writing on the blackboard. Therefore, that solution cannot be achieved in the present educational environment.

**Figure 1:**
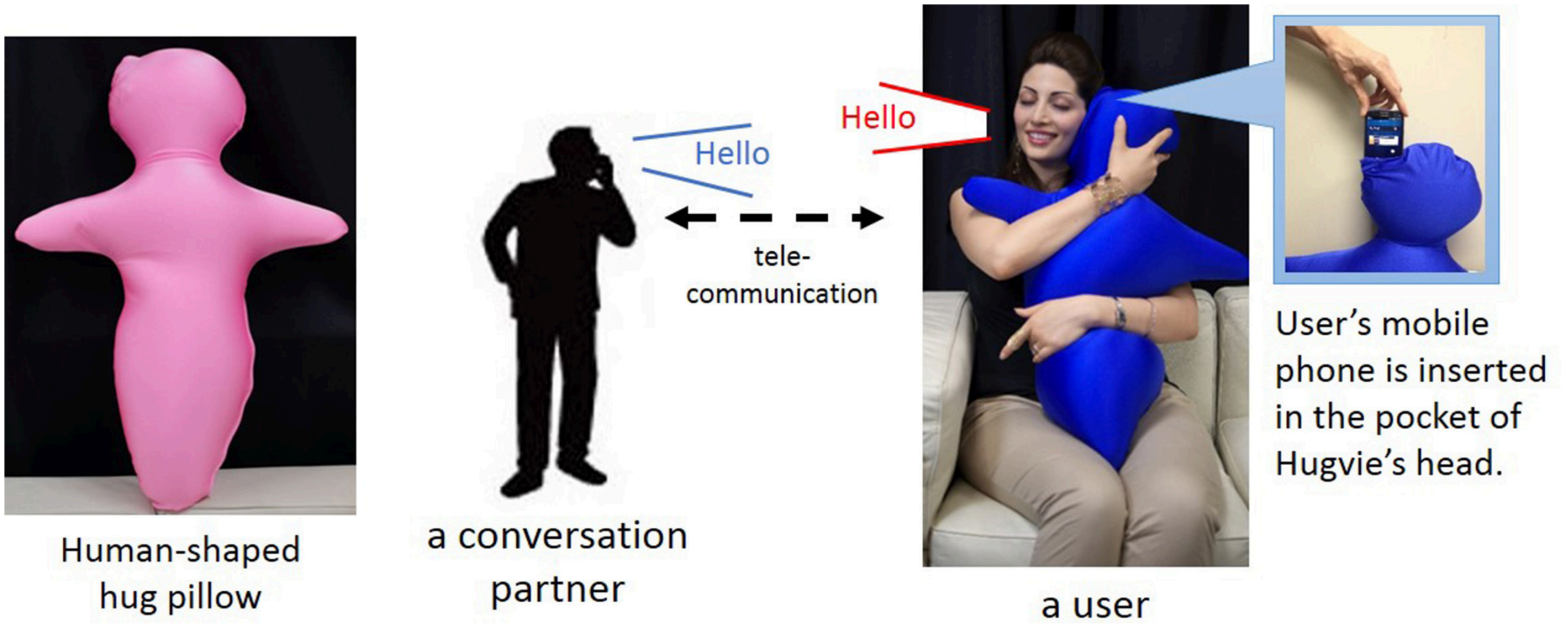
**Hugvie: huggable communication medium**.

We introduce a human-like communication medium as a proxy for teachers to achieve intimate social interaction in classrooms and support both forming external information and preparing mental states for listening. In this study, we use a huggable communication medium called Hugvie with which users can strongly experience the presence of remote partners while hugging it (Minato et al., 2013) (Figure 1). Hugvie, whose body is mainly a cushion in a human-like shape, allows users to feel as if they are hugging conversation partners by squeezing something human-like and hearing a voice near their ears. Since a previous study has already shown that conversation with Hugvie reduces stress (Sumioka et al., 2013), we expect that it will also help children prepare themselves for listening to others by improving both their external conditions and mental states.

However, since this is the first study that introduces a huggable communication medium into classroom activities, it remains unclear how children and educators will react to it and whether they will accept it. In this paper, we present three case studies where we introduced Hugvie in storytime settings and observed how children react to investigate whether it improves children's listening. We also investigated its acceptability by children and storytellers because acceptability to new information systems indicates their successful introduction into our lives (Nickerson, 1981; Gould et al., 1991; Davis, 1993). In particular, human-like devices might be rejected, as implied by the “uncanny valley” effect, which suggests that people have uncomfortable feelings to human-like robots as their appearances become more human-like (Mori et al., 2012). This effect is usually discussed in interaction between adults and very human-like robots. But one study implied that children do not exhibit positive responses to a robot with a more abstract human representation (Yamamoto et al., 2009). Furthermore, children may hesitate to hug such devices because they can feel their teacher's presence from Hugvie. Therefore, in this paper, we qualitatively and quantitatively investigate these two possibilities, the improvement of children's listening with Hugvie and social acceptance to Hugvie, through field observations and discuss supporting children's listening by a communication medium.

## 2. Materials and methods

### 2.1. Hugvie

Hugvie, a huggable communication medium, is a human-shaped cushion (75-cm high and 600 g) that was designed as a communication device to give users a hugging experience. It is a soft cushion filled with polystyrene microbeads and covered with spandex fiber. Putting a hands-free mobile phone inside a pocket of its head enables users to talk while hugging it (Figure 1), increasing the feeling they are actually hugging a distant conversation partner.

### 2.2. Storytelling system with Hugvie

We focused on storytelling as a typical activity since teachers spend more than half of their class time on verbal instruction from elementary school to college school in different countries (Janusik and Wolvin, 2009) and it is often used as a teaching tool for organizational learning and received wisdom (Haigh and Hardy, 2011). Storytelling in elementary schools is usually done in one-to-many communication; a storyteller reads a picture book to many children, while Hugvie is used in one-to-one interactive communication (e.g., Minato et al., 2013; Sumioka et al., 2013). Therefore, we applied radio broadcasting for one-to-many storytelling by putting a radio receiver inside Hugvie instead of a mobile phone.

**Figure 2:**
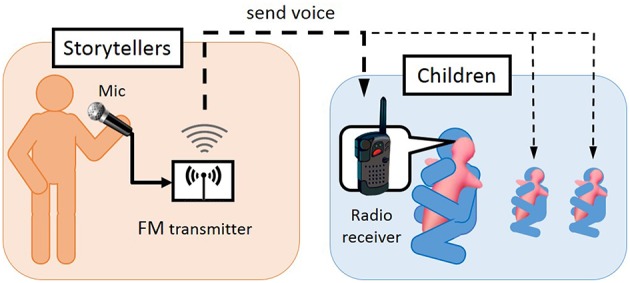
**Storytelling system with Hugvie**.

Figure 2 shows our radio broadcasting system for storytelling. Storytellers tell the child listeners a story by showing a picture book through a microphone connected to a FM radio transmitter. All of the children listen to the storyteller's voice near their ears through radio receivers while hugging their Hugvies. Note that children can also directly listen to the storyteller's voice since both are in the same room. However, they will probably feel that the storyteller is whispering to them since they simultaneously hear the storyteller both directly and through the radio receivers.

### 2.3. Case study 1: Introducing huggable communication media into general storytime for children

#### 2.3.1. Aim

For investigating the impact of a huggable communication medium on children's listening and its acceptability by children and teachers, we introduced Hugvie into a storytime activity and observed the responses of children and storytellers. Storytime includes just storytelling and one with using tools such as pictures, books, and toys. We observed storytime to allow us to get much information about children's listening because they are mainly listening during storytime. We conducted a field experiment to observe the natural responses of children and teachers to Hugvie.

#### 2.3.2. Subjects and procedure

Thirty-three preschool children who are 5 or 6 years old participated in a storytime event. This study was approved by the ethics committee of the Advanced Telecommunications Research Institute International (Kyoto, Japan). Since the subjects were young children, we explained this study to all the parents and received informed consent from them. We received permission from the parents and the school to include the image records of the children for research purposes. The child participants were given Hugvies at the school's library and shown the correct posture for using them by a male experimenter: sitting straight and hugging their Hugvie to enable a device at its head to contact the children's own ear (Figure 1). We confirmed that all of the children could hear the male experimenter's voice from Hugvie at a comfortable volume after adjusting the volume on the radio receiver inside each child's device. Female volunteers with much story-time experience did storytime for children. At the beginning, a volunteer did a few tricks and sang rhyming songs with the children for about 4 min to make sure that the children realized how Hugvie works. Then three other volunteers told them a story illustrated with picture cards for about 7 min (**Figure 4**). After another 3-min trick show, another story was told for about 11 min. We call these trials where the children used Hugvie the *Hugvie condition*. After that, we collected the Hugvies from the children and two paper-cutting activities were performed for about 26 min (**Figure 5**), where two different volunteers told two stories while cutting colored paper and combined them into the characters and the scenery from the stories (*typical condition*). Finally, all the children sang while a volunteer played the piano.

#### 2.3.3. Measurement

Two coders who did not know the purpose of the experiment analyzed the recorded movies to identify the behavioral differences between the typical and Hugvie conditions. Since the time between the two conditions was different, we used the first 25 min of the movies in each condition. Children sometimes moved beyond the video camera or overlapped with another child since they were more active than we expected in the typical condition. We eliminated their data from further analysis when at least one of the coders had difficulty judging their face directions. After this preprocessing, the data collected from some children became much smaller than in the storytime because they disappeared many times from the video camera. Therefore, we used the data collected from 29 children who were observed more than the 75 percent of the whole movie in each condition for our analysis.

We defined not listening to the storytellers as children who did not direct their faces toward the storytellers as captured from the movie data. The coders coded whether each child listened to the storytellers on a second-by-second basis through the movies. The inter-coder agreement score through the used data was κ = 0.62, indicating substantial inter-observer reliability (Viera and Garrett, 2005). We calculated the not-listening rate (NLR) for each child in each condition to evaluate the behavior of the children with the data where both coders agreed on child (not) listening: *NLR* = *NL*∕(*NL* + *L*), where *NL* indicates the total not-listening time and *L* is the total listening time.

#### 2.3.4. Results

**Figure 3:**
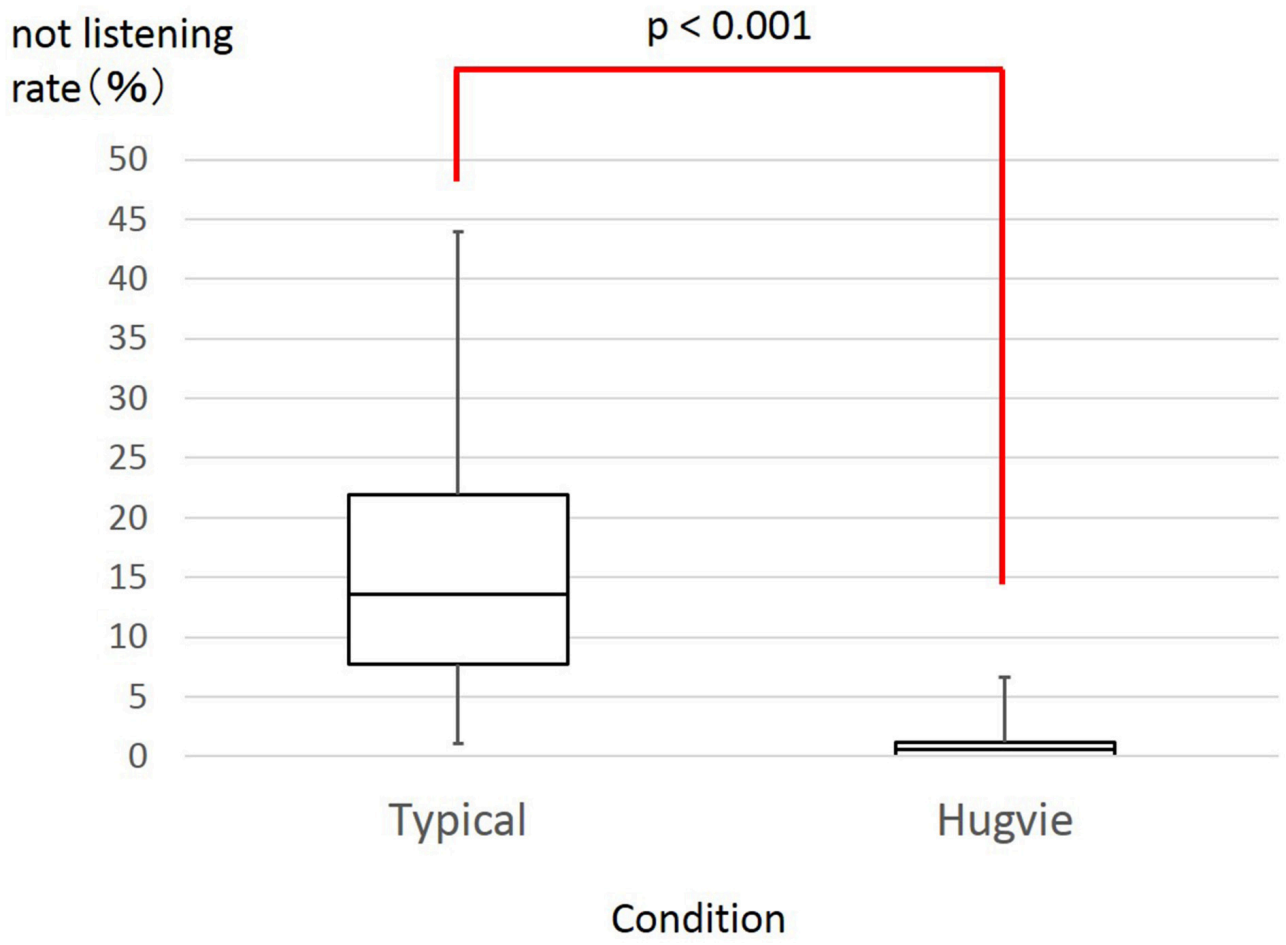
**Rate of children who directed their faces at something other than volunteers**.

**Figure 4:**
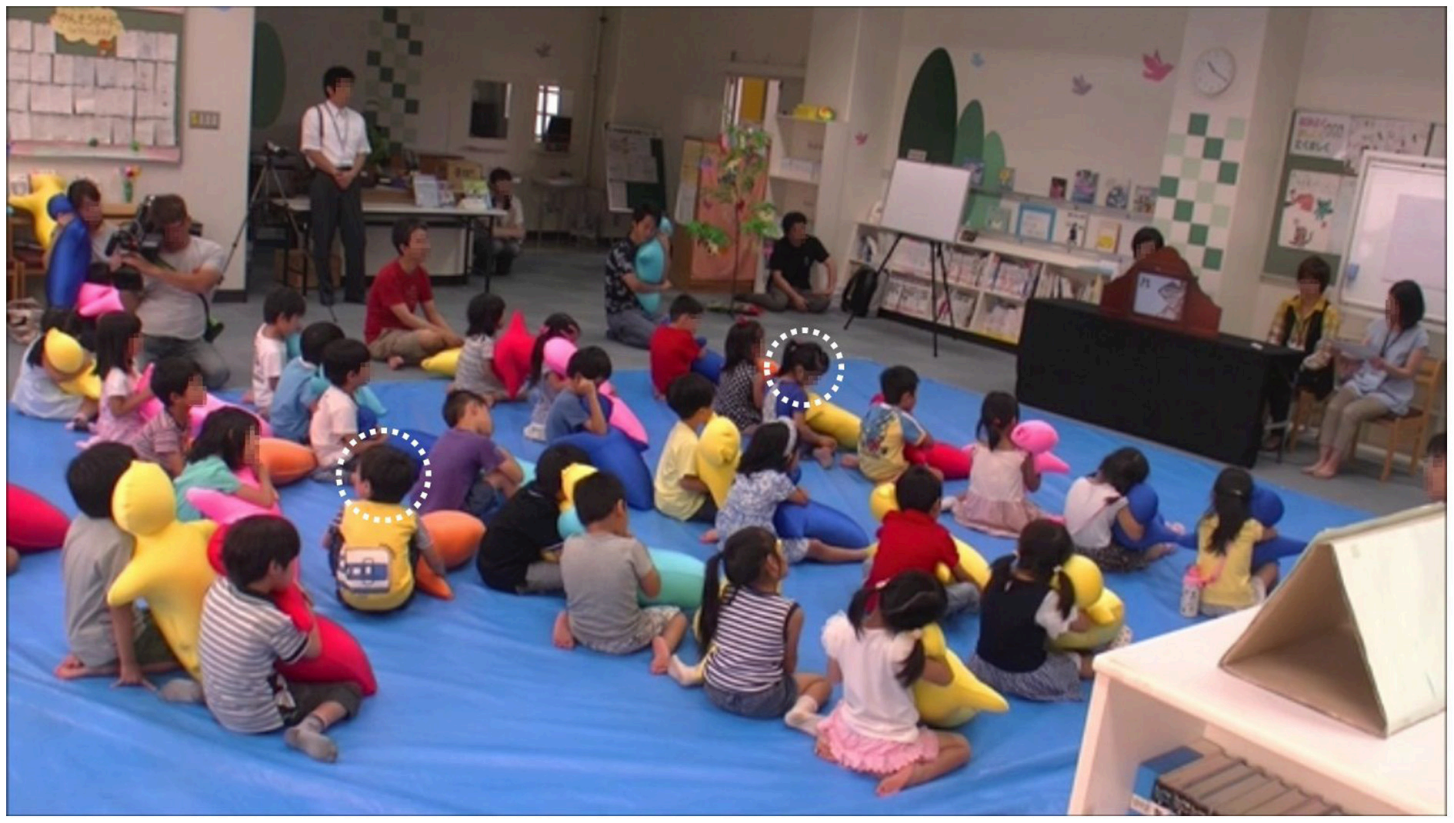
**Storytime with Hugvie (12 min. later): two of 30 children (total number countable from this figure) became distracted (white dotted circles)**.

**Figure 5:**
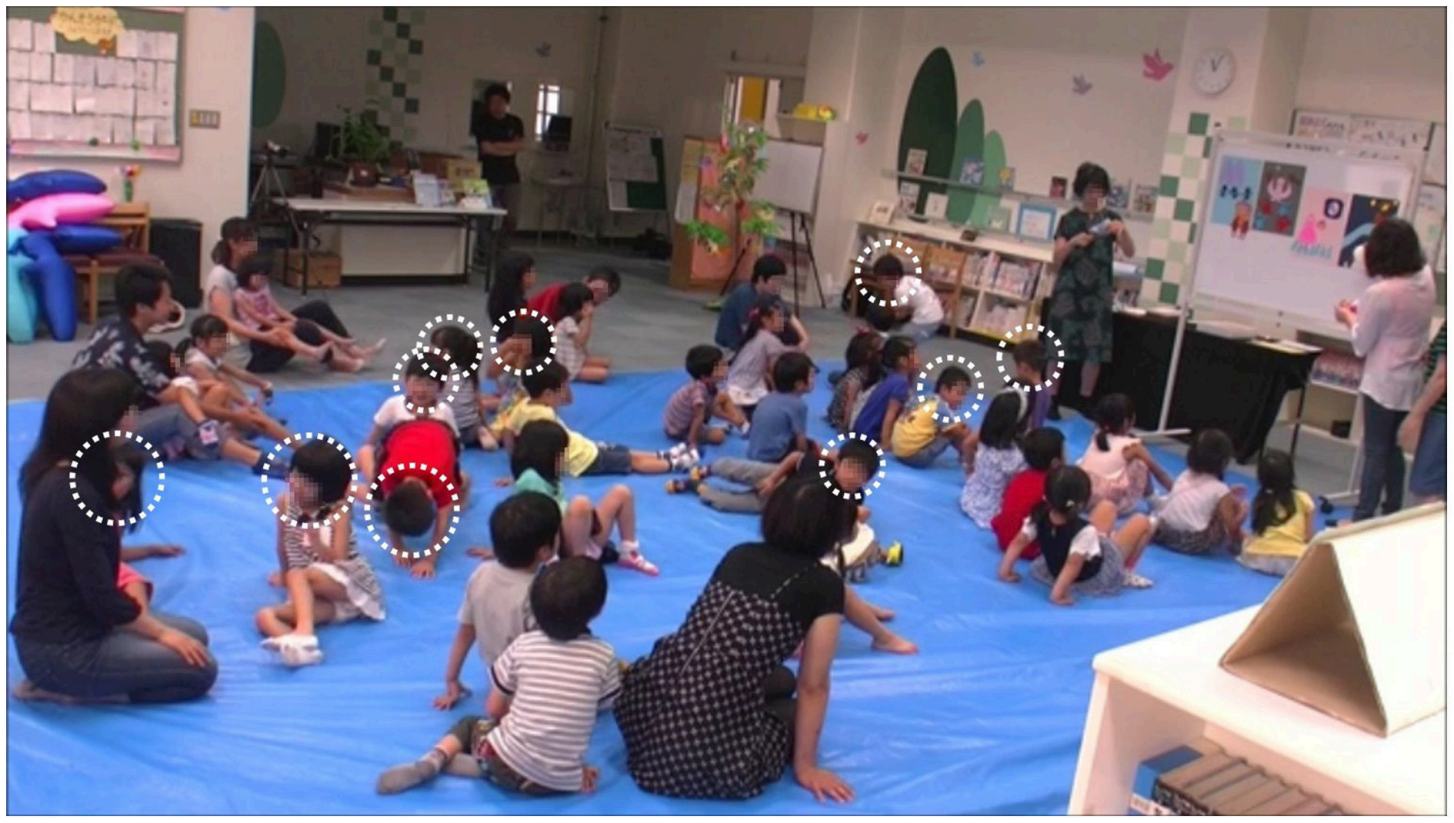
**Typical storytime (57 min. later): 10 of 30 children became distracted (total number countable from this figure) (white dotted circles)**.

Hugvie produced big changes in the children's behaviors. Figure 3 shows the listening scores in the typical and Hugvie conditions. We found significant differences between them with a paired *t*-test (*t* = −6.83, *p* < 0.001, ES: *d* = 1.27). Figures 4, 5 show the typical behaviors of children whose attention was drawn to something else in the two conditions. In the typical condition, some children walked around the room and talked or played with others after losing interest in the storytellers. The children who were far from the storytellers tended to engage in such behavior. On the other hand, such behaviors did not occur when children used Hugvie, although a few children looked away from the storyteller. Interestingly, the children at the back of the room seemed to listen to the volunteers' voices from Hugvies without any complaints even though they had difficulty seeing the picture cards.

No children rejected or showed dislike of Hugvie, though some children did not use it.Two children who did not understand how to use it were helped by volunteers, and some near the storytellers listened directly to the story instead of through their Hugvies. They seemed to feel comfort and fun from Hugvie. For example, some children said “*It really feels good!*” when they hugged their Hugvies. One girl in the back also appeared to be having fun during storytime and expressed her feeling to an experimenter.

The teachers and volunteers who observed the event were surprised at the result. A female volunteer said, “*I'm really surprised that Hugvie easily calmed the children because we usually spend lots of effort relaxing the children and keeping them calm so that they can pay attention to the story. I want to introduce Hugvie into other activities like storytime to toddlers or elderly people*.” Two other volunteers made similar comments.

#### 2.3.5. Discussion

We found that listening through Hugvie decreased the number of children who didn't seem to listen. While children were often distracted during typical storytime, children with Hugvie paid more attention to the storytellers. This effect appears stronger for children in the back of the room since they tend to lose focus without Hugvie due to their distance from the storyteller. On the other hand, the closer the children are to the storytellers, the weaker this effect might be since some children near the storytellers listened without their Hugvies.

Children showed no negative impressions toward Hugvie. Rather, they often expressed positive impressions such as comfort and fun. Note that the children accepted Hugvie not only in the storytime sessions by the female volunteers but also in instruction about it by a male experimenter. Perhaps, Hugvie is basically accepted by children in storytime regardless of the gender of adult storytellers.

The educators and the volunteers were also surprised at the changes in the children. This implies that the introduction of Hugvie is useful in school education. One teacher suggested that Hugvie was cast as a proxy of the storyteller: “*Basically, the students are listening to their teacher in a one-to-one conversation even though some have difficulty focusing on their teacher in class. Listening through Hugvie might enhance their feeling of a storyteller who's talking directly to them*.” A volunteer pointed out a change in her storytime: “*I concentrated on reading the book since I didn't need to read loudly so that the children in the back could hear me*.” Storytime with Hugvie might facilitate children's listening by allowing storytellers to devote more concentration on telling a story.

Hugvie showed the potential of a huggable communication medium to facilitate children's listening. However, we need more trials to test its effects because this case study is not a perfect comparison; the two environmental conditions are different. For example, storytime with Hugvie was done before the typical condition. Children might be nervous because they have few experiences of being in school, so that they might not talk and play in the former condition. Another difference is that the length of the concentration required in typical storytime is longer than with Hugvie because it is hard for children to maintain concentration for a long time. Since the rest time is also less in the typical condition than in the Hugvie condition, children might be so tired that they became easily distracted in the latter. The storytime contents were also different. For storytime without Hugvie, the volunteers often said nothing while cutting paper. Such a boring time might cause children to lose interest in the storytime. However, given the fact that volunteers with much storytime experience felt surprised by the children's behavior, Hugvie might still positively impact listening. Such surprises reflected the children's changes from more than just a few of them who didn't listen.

Practically, storytime styles vary in certain situations and such differences might change the listening support effect. For example, various persons can be storytellers. In this case study, the storytellers were mainly women with much storytime experience. Their expertise might induce Hugvie's effect. Can amateur storytellers promote the Hugvie effect? Another example is a group activity that is often performed as a class activity. While only one story was told to children at the same time in this experiment, members of different groups tell different stories to other group members in parallel. In such a situation, children have to listen to their storyteller in a noisier situation than in this experiment. Can Hugvie still support children? To address these questions and investigate how different storytime situations affect Hugvie's supportive effect, we introduced it into storytime by child storytellers as a group activity in case study 2.

### 2.4. Case study 2: Introducing huggable communication media into simultaneous storytime in children groups

#### 2.4.1. Aim

To investigate whether Hugvie encourages children to concentrate on listening in such noisier situations as group activities, we introduced it into simultaneous storytime in children groups as a different storytime style from case study 1. Since most children's speaking skills are less advanced than those of adults, casting a child as the storyteller can investigate whether, regardless of a storyteller's speaking skills, Hugvie prompts listening. Additionally, we set at most four storytime groups at the same time to observe Hugvie's effect in a noisier situation. Such an investigation is valuable not only because it is the first such trial of a huggable communication medium but also because it is more difficult to pay attention to a story without Hugvie in those situations; lesser speaking skills disturb precise listening comprehension, and in simultaneous storytime groups, storyteller voices offset each other.

#### 2.4.2. Subjects and procedure

**Figure 6:**
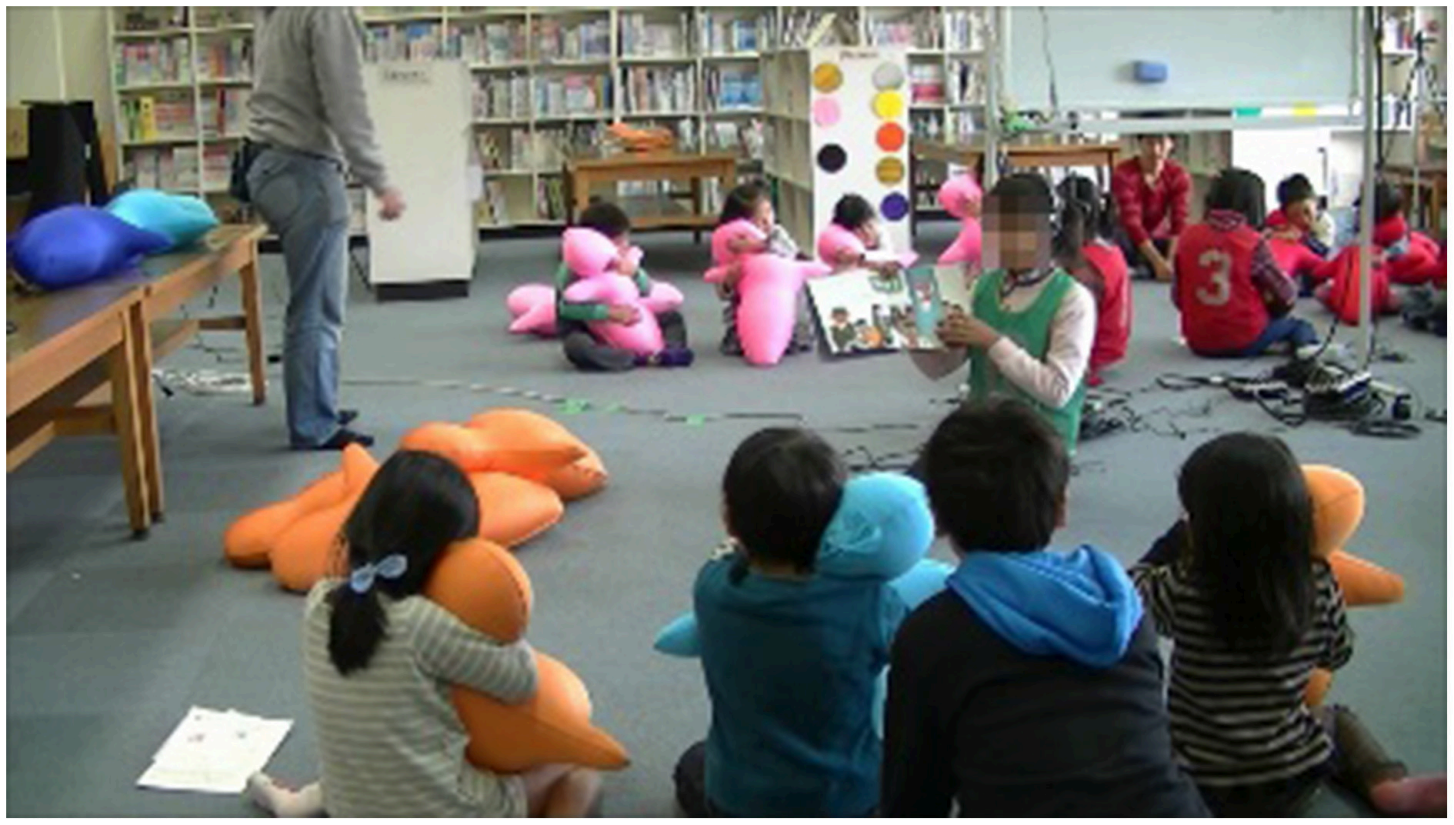
**Simultaneous storytime in children groups**.

We introduced Hugvie into storytime sessions in the elementary school event to 139 preschool children who are 5 or 6 years old. They were divided the children into 34 groups of three to five kids with two or three 5th graders as guides of the school. Each group could freely join several sessions (including storytime) in the event. This study was approved by the ethics committee of the Advanced Telecommunications Research Institute International (Kyoto, Japan). We explained our study to all of the children's parents and received informed consent from them. In the storytime events at the school's library, they were given Hugvies and instructed how to use them by showing the correct posture as described in case study 1. We confirmed that all children could comfortably hear the experimenter's voice from their Hugvies. After that the 5th graders told stories with picture books to the preschool children for 10 min (Figure 6). At most four groups of storytime were held at the same time in the same room. The other groups waited in the room until some of the four groups had finished and moved on to other events.

#### 2.4.3. Measurement

We video-taped the storytime sessions and observed the children through the recorded movies. We received permission to include image records of the children from their parents and the school for research purposes. We categorized the children who did not direct their faces to the storytellers as children who did not listen to the story.

#### 2.4.4. Results

As the event continued, the room got louder owing to the children who were waiting to join the storytime with Hugvie. Some children chased each other around the room, and others played and/or talked with their friends or their fifth-grade guides. A few waited in silence. However, most children concentrated on the listening to the story in silence once they joined the Hugvie storytime session. Only 6% sometimes lost their attention, but they soon resumed listening without walking around or talking with others. No children rejected Hugvie. They showed such positive impressions as looking comfortable, as we observed in case study 1 when they held Hugvies.

#### 2.4.5. Discussion

Our results showed new potential applicable occasions for Hugvie. Regardless of the low speaking skills of the 5th grade storytellers, the preschool children listened with Hugvie. This means that anyone can be a storyteller regardless of speaking skills.

This result also suggests that Hugvie reduces not only impediments in the listening process but also the requirement needed for speaking. Although the experiment room was quite noisy due to simultaneous storytime and children who were waiting to join storytime sessions, they concentrated on listening with Hugvie. Hugvie enabled us to hold storytime in noisy environments because it produced the speaker's voice near the user's ears and relaxed the children. This achievement is completely different from what was reached by a listening support device, such as the sound field amplification system, because such devices drown out other sounds in the entire room by amplifying the speaker's voice.

**Figure 7:**
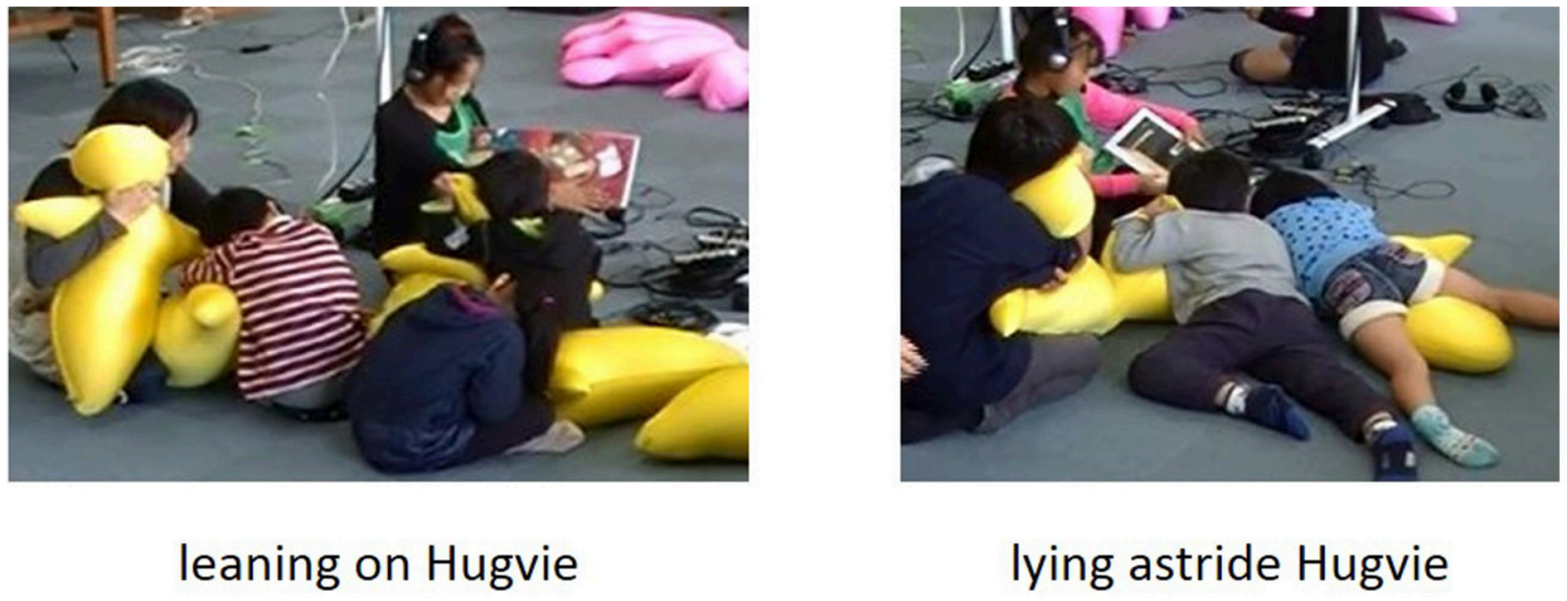
**Two listening behaviors**.

We did find one negative aspect of storytime with Hugvie with respect to body posture from recorded movies. As the storytime continued, a few children showed incorrect postures, although they were correctly holding Hugvie at the beginning of the storytime: leaning on or lying astride it. While 83% held Hugvie as instructed by the experimenter, 10% leaned on Hugvie and 7% lay astride it (Figure 7). This might be a problem for its introduction into school education because posture is important for health management related to physical development and visual loss (Kratěnová et al., 2007). Therefore, we need to improve Hugvie to prompt children to maintain good posture. Our observation suggests that its softness caused bad posture. Since Hugvie is easy to bend and fold, children sitting on the ground tended to bend their backs and lie on Hugvie.

As with case study 1, none of the 139 children rejected Hugvie. However, this does not mean that all of the children were pleased with it. Some might have used it because the adults asked them to do so. There is room to investigate acceptability; performance may fall if children are unwilling to use a device. Since previous introductions of support devices into schools (Tanaka et al., 2013; Komatsubara et al., 2014) showed the importance of willingness to use, in case study 3 we asked the children whether they are willing to use Hugvie after storytime with it.

### 2.5. Case study 3: Willingness to use huggable communication media

#### 2.5.1. Aim

For investigating how willing children are to use Hugvie, we gave them the option of using it or not in storytime after they and their parents experienced storytime with Hugvie once. Observing whether children used it in that situation shows their willingness to use it. We also asked the children about their impressions of Hugvie.

#### 2.5.2. Subjects and procedure

We introduced Hugvie into storytime for children at a science museum in Tokyo called Miraikan. Our participants, 29 children and their parents, were gathered in an open space of the museum by its staff members who explained the event. This study was approved by the ethics committee of the Advanced Telecommunications Research Institute International (Kyoto, Japan). We explained this study to all the parents of the subjects and received informed consent from them.

The experiments consisted of two sessions: forced and free. In the forced session, the participants were divided into two groups by families, and one group was given Hugvies and instructed how to use them. After we confirmed that all children could comfortably hear Hugvie's voice, a female volunteer with much storytime experience with children read a story with a picture book. Then we collected the Hugvies and gave them to the other group, and the volunteer told a story with an another picture book. Each storytime session lasted about 5 min.

**Figure 8:**
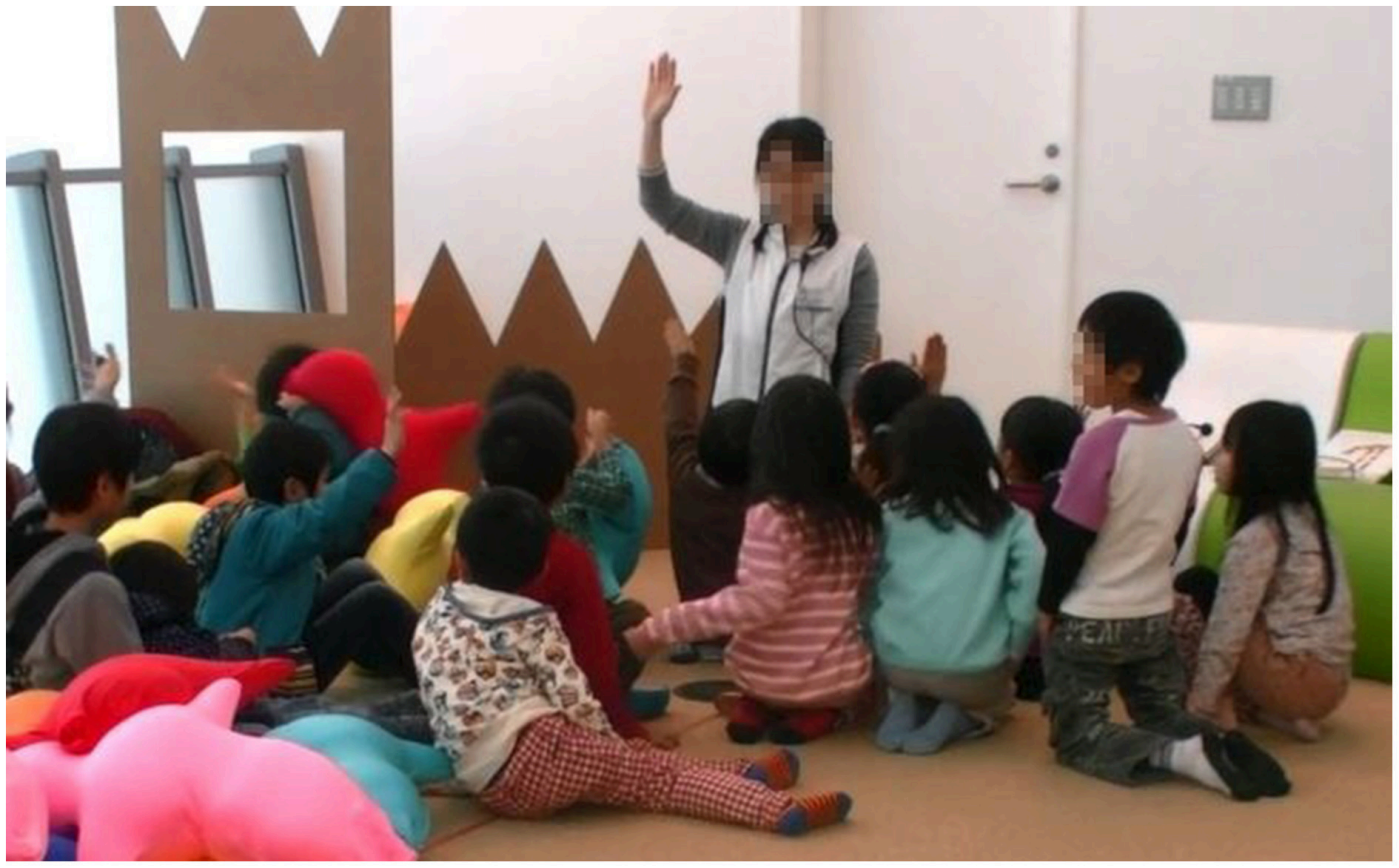
**Asking children whether they want to use Hugvie after storytime**.

After the forced session (storytime with parents) finished, a free session was conducted. A female staff member of the museum gathered only the children and asked them whether they wanted to use Hugvie for another storytime session (Figure 8). She also asked them to express their thoughts about Hugvie. Then she read another book with/without Hugvie according to their own willingness to use. During the free session, an experimenter explained the purpose of the experiments and studies with Hugvie to their parents in the back of the area and asked them by a questionnaire for their impressions about their children using Hugvie. The experiments were recorded. The event was held twice: 15 children participated in the first event and 14 in the second. The child participants ranged in age from 3 to 10.

#### 2.5.3. Measurement

We counted the number of children who used Hugvie in the free session of each event and also checked their impressions of it in the free sessions. We collected comments from 21 parents about their impressions of their children using Hugvie. Two coders who did not know the purpose of our experiment read all the comments and categorized the parent impressions of children using Hugvie as positive, negative, or neutral. The inter-coder agreement score was κ = 0.83, indicating almost perfect inter-observer reliability (Viera and Garrett, 2005). In addition, we extracted the behavioral differences of the children between storytime with and without Hugvie in the forced session through the recorded movies. We received permission to include the image records of the children from their parents and the museum for research purposes.

#### 2.5.4. Results

When we asked children whether they wanted to use Hugvie, six of 15 and seven of 14 used Hugvie in the first event. In interviewing the children in the second event, the children who were pleased with it made such comments as, “*Using it allowed me to listen more clearly*” and “*It's so cute*.” On the other hand, the children who were unwilling to use it commented that “*It's difficult for me to hug it and listen*” and “*Hugvie's voice was so loud that it gave me a headache*” (Table 1).

**Table 1:** **Number of children who willingly used Hugvie and the reasons of their decisions**.

	**Willing to use**	**Unwilling to use**
Number of children	13	16
Reason	Able to listen clearly	Difficult to hug and listen
	Hugvie is cute	Too noisy
		Hugvie is not cute

The results of the parents' impressions showed that more parents had positive impressions than negative. Twelve felt Hugvie had a positive effect on their children. Eight of 12 recognized that their children concentrated more on listening to the story with Hugvie. One mother reported that her child seemed to come back to her to be comforted during the storytime session without Hugvie. Three others hoped to use Hugvie in kindergartens or while their children were alone at home. Seven parents showed negative impressions of Hugvie. One father said he did not notice any Hugvie effect on his child. One mother found that her child looked sleepy. Two parents were worried that their children would get bored with Hugvie, and three parents wanted the interface to be improved, such as the sound quality and ease of use. The rest of the parents reported partial positive impressions of Hugvie for its usefulness for children who are far away from the storytellers although one of two coders categorized their impressions as negative or neutral.

We found some interesting behaviors of the children in the forced sessions. During storytime, nine ran up to and grabbed their parents when they were not using Hugvie, although they did not do that while using it. Two children with slightly smaller bodies than Hugvie repeatedly quit paying attention to a storyteller regardless of the conditions, and the other children almost always concentrated on listening to the storytime in both conditions.

#### 2.5.5. Discussion

Approximately half of the children were willing to use Hugvie, which means that a fair number of them were attracted to it after using it just once. It remains unclear whether the device's rate is high enough to introduce it into schools because no studies exist on the educational applications of similar communication devices. However, the rate is important as a baseline to improve Hugvie in respect to willingness to use it.

Our interviews and questionnaires showed that many children and their parents felt that Hugvie prompted users to concentrate on listening. In other words, Hugvie had such a strong effect that users noticed the difference caused by it. On the other hand, a few users did not feel any effect. We infer that this was mainly caused by the interface problems, including unsuitable size, sound quality, and/or ease of understanding how to use it. For example, Hugvie requires users to place their ears near the speaker because it is not very loud. Thus, misunderstanding the speaker location prevents adequate listening to the story through Hugvie, reducing its effect. These findings are important for improving Hugvie and the design policy of such support devices for telecommunication and physical interaction.

Children often run to and grab their parents, suggesting a desire to reduce their feeling of loneliness by making physical contact (Gallace and Spence, 2010). However, after using Hugvie, the children did not rush to greet their parents. We infer that this shows that using Hugvie reduced their feeling of loneliness the children felt during the storytime. If their parents were not near them when they were not using Hugvie, they would be distracted away from the storytime. Perhaps Hugvie encourages listening by improving not only the external condition but also the internal condition. On the other hand, two young children did not listen calmly in either storytime condition. Their bodies were too literally small to use Hugvie. In this case, its unsuitable size disrupted its use and reduced its effect.

### 2.6. General discussion

Out of the 201 children in all the case studies, none rejected our huggable communication medium, which suggests Hugvie might be accepted by most preschool children. Yamamoto et al. reported that 2- to 3-year-old children did not exhibit positive responses to a small robot with a non-human-like appearance that showed human-like contingent actions. They argued that perhaps the children experienced the uncanny valley effect due to the conflict between the robot's appearance and its human-like actions (Yamamoto et al., 2009). Although Hugvie has such an intrinsic conflict between its abstract human form and a human voice from its inner communication device, our results suggest that it does not produce negative feelings in children. Schools might be receptive to introducing huggable communication media into their curriculums.

However, not all of the children were satisfied with Hugvie. Some were unwilling to use it due to the difficulty of hugging and listening. Observations and user opinions suggested that the difficulty was caused by Hugvie's usability, including its size and stiffness, sound quality, and/or user-friendliness. This feedback provides insights into ways to improve Hugvie and highlights future research issues to be addressed before we introduce it into school education.

Case studies 2 and 3 suggested that such physical features as stiffness and size must be suitable for users. In case study 2, we found a potential problem when children use improper listening postures with Hugvie. Adult users never showed such postures since Hugvie was designed to be suitable for them. Children lean on Hugvie for support due to the immaturity of their musculoskeletal systems while adults can maintain their posture by themselves. We will verify our inferences in the future using another version of Hugvie that is stiff enough to support a child's body.

As reported in case study 3. Hugvie distracted children from listening if its size is inappropriate since small children with smaller bodies who had difficulty holding Hugvie often became distracted away from the storyteller. Another possible reason is that such distraction is caused not by a size mismatch but age. Younger children lacked the ability to sustain attention for a long time. Therefore, interesting future work might investigate the influence of size mismatch between users and Hugvie for listening with a smaller type of Hugvie.

The interviews and questionnaires of case study 3 also suggest that some users could not listen well with it because they did not understand how to use it. We need to improve Hugvie's interface to reduce such future misunderstandings. For example, marking where users should place their ears is a possible improvement. An automatic volume control system while holding Hugvie would allow each user to adjust Hugvie's volume.

As reported in case study 1, the children near the storytellers attentively listened without holding Hugvie because the storyteller's voice was louder than the sound from Hugvie. Perhaps Hugvie's voice should be the strongest stimuli among the surrounding sounds, including the storyteller's direct voice, to encourage children to use Hugvie. Although children do not need to use it when they are near a storyteller, they might benefit from using it in other aspects, as suggested in studies on interpersonal touch. For example, a brief touch from teachers motivates children to participate in lessons (Guéguen, 2004). We expect similar effects on children when they are holding Hugvie. Tactile stimulation from it would encourage the voluntary behavior of children when they listen to a teacher's request through Hugvie. Further investigation is needed.

We also found evidence that Hugvie might benefit both teachers and children. In case study 1, as pointed out by a volunteer, the storytellers concentrated more on the story's content with Hugvie since they did not need to speak so loudly. Previous studies report that teachers often suffer from such voice problems as phonation difficulties, hoarseness, and throat pain because they have to speak loudly to control their classrooms (Yiu, 2002). Sound field amplification systems provide a possible solution to this problem. However, increasing the sound volume in a classroom might disturb the class in the next classroom if rooms are not properly soundproofed. On the other hand, Hugvie reduces the noise level in class and improves the external conditions because it enables teachers to talk in a lower voice and children to concentrate on listening in class. Therefore, Hugvie helps reduce the voice problems experienced by teachers and enables them to concentrate on improving their teaching.

Our results also show the possibility of Hugvie's future applications. We found that our proposed storytelling system enables children to become immersed in a story even with an inexperienced storyteller in noisy environments. We also expect that Hugvie can be introduced into other activities, such as interaction with senior citizens and group work. Interaction between children and seniors is difficult because the listening skills of the latter are often poor and children's speaking skills are immature. The results of case study 2 tell us that Hugvie can deal with both problems.

Group work requires concentration on conversation among the group members. However, usually some voices are drowned out by other group conversations. Hugvie's vocal sounds can overcome the surrounding conversations without offsetting them. It can also evoke interest in a speaker (Nakanishi et al., 2013), indicating that it encourages the involvement of each member in group discussions.

Although all of our case studies suggest that a huggable communication medium has a possibility to support children's listening skill, further investigation is needed. First, we have to evaluate Hugvie's effect on children in more controlled conditions. Another important issue to be addressed is the investigation of how deeply Hugvie affects cognition. In this paper, we focused on the changes in the children's behaviors and social acceptability to Hugvie since this is the first study that introduced a huggable communication medium into educational situations. However, perhaps listening through Hugvie enhances information comprehension and memory more than usual listening. Actually, such enhancements are needed in education. Many graduate school students of college have high listening skills (McDevitt et al., 1991), and most college students who fail examinations lack listening skills (Conaway, 1982). As a next step, we have to verify a story's comprehension with some sort of listening comprehension quiz.

## 3. Conclusion

Through three case studies, we demonstrated that huggable communication media show possibilities to encourage children to listen to others. Our huggable communication medium, Hugvie, virtually enables intimate interactions with conversation partners to improve external and internal conditions for listening. Our results showed that Hugvie, which addressed the classroom problem where children did not listen to a speaker, is accepted by children, their caregivers, and their educators. Our results also suggest that Hugvie can support communication between people who sometimes suffer from low speaking skills and low listening skills, such as children and seniors. We hope the intimate interactions mediated by huggable communication media can reduce problems of school education and other situations where listening skills are crucial and encourage people to learn from others.

## Author contributions

JN and HS wrote the main manuscript text. All authors designed research.

### Conflict of interest statement

The authors declare that the research was conducted in the absence of any commercial or financial relationships that could be construed as a potential conflict of interest. JN declare no potential conflict of interest. HS and HI are employed by ATR. ATR has patents on Hugvies. HI has consulted for Vstone Co., Ltd., which sells Hugvies, and received compensation. He also owns stock in the company.
